# Extending the Together, We Inspire Smart Eating Curriculum to Intergenerational Nutrition Education: A Pilot Study

**DOI:** 10.3390/ijerph19158935

**Published:** 2022-07-22

**Authors:** Rachel M. Scrivano, Jill J. Juris, Shannon E. Jarrott, Jennifer M. Lobb

**Affiliations:** 1 The College of Social Work, The Ohio State University, Columbus, OH 43210, USA; jarrott.1@osu.edu; 2 Beaver College of Health Sciences, Appalachian State University, Boone, NC 28608, USA; jurisjj@appstate.edu; 3 College of Food, Agricultural, and Environmental Sciences, The Ohio State University, Columbus, OH 43210, USA; lobb.3@osu.edu

**Keywords:** implementation, intergenerational, intervention, nutrition education, preschool, mixed methods

## Abstract

The COVID-19 pandemic has made accessing nutritious foods difficult for older adults and children living in low-income households. The evidence-based preschool nutrition education curriculum *Together, We Inspire Smart Eating* (WISE) can be used to encourage children to try healthy foods. Written as a single generation curriculum, inviting older adult community members to WISE programming for an intergenerational experience may provide further supports and mutual benefits as participants cooperate towards a common goal. While creators have evaluated implementation of WISE, research has yet to explore factors that influence WISE adoption within an intergenerational setting. We conducted a pilot study using the implementation evaluation framework to explore WISE implementation within single generation and intergenerational settings by measuring five implementation outcomes (fidelity, acceptability, appropriateness, feasibility, and sustainability) through three methods: (1) direct assessment of program fidelity via video coding; (2) indirect assessment of stakeholders’ perceptions of WISE implementation, and (3) a directed qualitative content analysis on annual interview data. Fidelity scores were comparable between the two settings and stakeholder ratings of appropriateness, acceptability, and feasibility of WISE were high. Qualitative data revealed that aspects of WISE are less appropriate for older participants and reiterated known logistical barriers of intergenerational programming that may challenge program sustainability.

## 1. Introduction

Eating habits formed during early developmental stages can impact a child’s taste preferences and eating habits in later life [1]. Parental influences and feeding strategies used within a child’s home are the primary determinants of life-long eating habits [2]. However, parents’ impact on children’s eating habits and food preferences may be influenced by social norms. A meta-analysis found that the most significant predictor of food intake was the presence of other people due to their modeling behaviors [3]. These social influences are well supported by social cognitive theory [4], which states that social contexts contribute to learning as social interactions, the environment, and others’ behaviors influence one’s own behaviors.

Preschool classrooms are a critical social setting in which nutrition interventions can be implemented. Programs such as Head Start provide children with a unique opportunity to socially engage and learn with others. Specifically, Head Start students spend 33 hours a week in childcare [5] where they are served nutritious meals throughout the school year [6]. Although Head Start centers adhere to the Dietary Guidelines for Americans [7], preschool-aged children have heightened food fussiness and food neophobia [8] that may reduce their intake of necessary nutrients [9]. However, children can become open to trying new foods [7] and learn to prefer unfamiliar foods through repeated exposure [10]. As supported by a meta-analysis, repeated taste exposure interventions can promote vegetable intake among preschoolers [8]. Such interventions are particularly important for children in low-income households who may not have access to try and consume nutritious foods due to experienced food insecurity [11,12]; interventions can lower their risk for diet-related chronic diseases [13] and overall poor health [11], providing them an opportunity for a healthier adulthood.

*Together, We Inspire Smart Eating* (WISE) is an evidence-based nutrition education program for high-risk preschool-age children who come from low-income families and/or have resource-poor backgrounds [14]. The WISE curriculum centers around sensory food experiences facilitated through educator training, classroom curriculum, and educational materials for parents. During classroom-delivered Discovery Units, children engage in food experiences that allow them to explore cost-effective and readily available fruits and vegetables. Importantly, educators model appropriate behaviors (e.g., eating the target foods) to encourage the children to try healthy foods [15]. In recent studies, WISE researchers demonstrated the curriculum’s effectiveness for increasing healthy food consumption; children involved in WISE programming consumed less sugary foods and more fruits and vegetables after completing Discovery Units [15,16]. Although WISE has been an effective nutrition program for at-risk preschool children, its use in other contexts has not been determined [14].

One potentially powerful extension of the WISE curriculum involves older adults participating alongside preschoolers. Similar to children, many adults over the age of 50 experience food insecurity [17]. Despite research estimating that food insecurity impacts 14% of US adults between the ages of 50 and 80 years [17], the COVID-19 pandemic exacerbated numbers experiencing food insecurity, especially among vulnerable populations [18]. Intergenerational programs, those that purposefully bring youth and older adults together for mutual benefits, may provide participants with positive social experiences while they pursue common goals and build community cohesion [19]. Thus, not only can older adults’ active involvement assist in fostering children’s acceptance of new foods by serving as an additional model [4], but such intergenerational programming may foster positive relationships between groups who may otherwise lack opportunities to connect, as supported by intergroup contact theory [20,21]. Although previous intergenerational nutrition efforts have been studied [22], implementation research of intergenerational programming is largely absent from the literature, presenting a unique opportunity with the current study [23,24]. 

Before understanding older adults’ influence on nutrition education delivered to preschoolers, research must first determine whether their inclusion is possible, that is, evaluating if the preschool WISE curriculum could be implemented within an intergenerational setting. Evaluation frameworks provide researchers with a descriptive checklist to guide the measurement of program elements believed to influence implementation [25,26]. One evaluation framework [27] depicts implementation based on outcomes of acceptability, adoption, appropriateness, costs, feasibility, fidelity, penetration, and sustainability. Researchers have assessed some of these implementation outcomes for WISE among preschoolers in past studies. For example, one study comparing educators’ self-assessment and researchers’ observations of WISE fidelity determined comparable ratings between the two assessment types [28]. Researchers also measured stakeholder and parental perceptions of acceptance, appropriateness, barriers, feasibility, and fidelity. Results of the study indicated high ratings of acceptability, appropriateness, and feasibility [29]. However, research should re-evaluate the implementation constructs [27] within the context of intergenerational programming given modifications made to program protocol.

Due to the novelty of integrating an intergenerational component to WISE, Proctor and colleagues [27] would suggest that certain implementation outcomes be measured early, including acceptability, appropriateness, feasibility, and fidelity. Therefore, we aimed to assess whether WISE programming could be successfully implemented within an intergenerational setting to extend its nutritional and social benefits to preschool students and older adults. The present study sought to answer the following research questions: (a) Is program fidelity of the WISE curriculum comparable across single generation and intergenerational settings? and (b) What are stakeholders’ perceptions of: (a) acceptability, (b) appropriateness, (c) feasibility, and (d) perceived sustainability of the WISE curriculum in a single generation and intergenerational setting? Therefore, this paper seeks to assess the implementation of the WISE curriculum and expand its application to practice within an intergenerational setting.

## 2. Materials and Methods

### 2.1. CBPAR and WISE Programming Procedures

Food for a Long Life (FFLL) was a five-year (2016–2021) USDA/NIFA Children, Youth, and Families at Risk (CYFAR) Sustainable Community Project [23,30]. This project was guided by nine principles of Community-Based Participatory Action Research (CBPAR) [31], which include the core components of involving partners in all phases of the iterative and cyclical research process, utilizing strengths and resources within the community to foster co-learning and capacity building, creating a balance between knowledge building and implementing intervention related to relevant and identified health problems, and working to inform intervention sustainability. Therefore, CBPAR provides a framework for addressing complex problems requiring constant communication and collaboration with community stakeholders [32]. FFLL goals were to increase healthy food knowledge, consumption, and access using intergenerational strategies among preschoolers and older adults in one Ohio and one Virginia community identified as food insecure. FFLL implemented WISE within two preschools and two adult day service centers in a Virginia community characterized as having the state’s highest rate of low-food access [33].

Aligned with a CBPAR approach, the FFLL team reviewed and selected potential nutrition curricula with key community stakeholders (e.g., preschool directors, preschool teachers, and adult day services staff). The group collectively decided to implement WISE programming in years four and five of the project. Although developers intended for teachers to deliver WISE in classrooms [14], FFLL utilized a roving model to accommodate personnel constraints and create intergenerational settings. The roving model required a trained FFLL team member to deliver WISE programming by moving between the two sites in a total of five preschool classrooms and two adult day centers. This FFLL team member attended a six-hour synchronous online WISE training session and adapted programming to the roving model [14]. Children participated in two WISE lessons monthly. Relying on teacher support and involvement, two preschools (four classrooms) and one adult day center participated in data collection for the present study. Two of the classrooms joined both single generation and intergenerational WISE programming, whereas the other two classrooms received only single generation WISE programming. Programming occurred during the academic year from September 2019 to the beginning of March 2020 (prior to COVID-19 social distancing restrictions). A description of activity programming follows. This project was approved by The Ohio State University’s Institutional Review Board (2016B0355) before programming and data collection took place.

For intergenerational WISE lessons, a FFLL team member would arrive at the adult day center and set up materials in a small activity room separate from where the full group of older adults was gathered. Chairs and materials were arranged in a circle around the perimeter of the room with markers at alternating seats to distinguish where children and older adults should sit. A small number of older adults were then invited to join the programming and move to this room. While they waited for the children, the FFLL team member described what they would be doing with the children during the activity (e.g., taste-testing a recipe using that month’s target food). Approximately 14 children and 2–3 teachers traveled to the adult day center by school bus and joined the older adults in the activity space. The FFLL team member then initiated the lesson by reviewing what the children had learned about the target food in a previous lesson, led an activity or game related to the target food, and engaged the group in a simple food preparation and/or tasting of the target food (e.g., peppers). The FFLL team member and attending staff helped the children and older adults prepare the food or respond to discussion prompts about the target food. Though not required, adults engaged in modeling for the children by trying the target food and making positive remarks about its taste or nutritional value. After cleaning up, the FFLL team member took the WISE mascot Windy the Owl puppet out of her cage to lead the group in a chant asking who tried the target food that day. Before departing the activity room, the FFLL team member asked the children to say goodbye to the older adults as they prepared to go back to the preschool. Activities usually lasted 30–35 min.

The present study focused on investigating two research objectives pertaining to the implementation of the WISE curriculum using both quantitative and qualitative methods. All participants (FFLL team members and site staff) provided informed consent prior to data collection. The following sections outline the methods used to answer each research question based on Proctor and colleagues’ framework [27].

### 2.2. Research Question 1

Is program fidelity of the WISE curriculum comparable across single generation and intergenerational settings?

#### 2.2.1. Method

The research coding team completed a direct assessment of WISE fidelity through standardized training with a Gold-standard observer (Dr. Taren Swindle) who co-developed WISE. Training consisted of a three-hour synchronous online session with instruction on: (a) intent of the 18 items comprising the WISE fidelity measure [28] (see Table 1); (b) differentiation of the fidelity measure’s anchors; and (c) application and interpretation of the fidelity measure across single and intergenerational settings. The coding team watched an example WISE lesson and discussed item scoring with the trainer.

To achieve interrater reliability, the four coders needed to reach agreement of 85% or higher on two occasions (i.e., two example videos) based on established ratings [28]. Specifically, identical scores or adjacent scores on the poles of the Likert scale (i.e., “1” and “2,” “3” and “4”) counted as agreement. The coders reached an interrater agreement of 86.11% on the first example video. Rating differences were discussed before coding the next example video. On two additional example videos, the team fell short of the recommended interrater agreement (76.38% and 72.00%); through discussion of the coding procedures with the trainer, coders reached 100% consensus on the videos.

Shifting from the WISE example videos, the research team next obtained interrater reliability from two FFLL videos (one single generation and one intergenerational WISE lesson). Using cutoffs of 85% for the 18-item fidelity scale [28] and 0.80 for Krippendorff’s alpha [34] calculated with ReCAL [35], coders achieved an agreement of 94.44% (α = 0.802) on a single generation setting video and a 97.00% agreement (α = 0.89) on an intergenerational setting video. Finally, two of the four trained coders individually coded five videos for a total of ten videos (six single generation and four intergenerational videos) recorded during WISE programming between October 2019 and March 2020.

#### 2.2.2. Measures

We adapted the 18-item fidelity measure [28] to assess fidelity of WISE programming. Though the roving model allowed for assistant facilitators (e.g., classroom teachers), coders only assessed the trained WISE facilitator. Items were measured using a four-point Likert scale that ranged from one (Not at all) to four (Very much). Unique anchors distinguished each item (see Table 1). Some items required reverse coding (see Table 1). Higher scores indicated greater levels of program fidelity, with scores that can range between 0 and 4.

#### 2.2.3. Analysis

Data were analyzed using SPSS version 25 (IBM Corp. Released 2017. IBM SPSS Statistics for Windows, Version 25.0. IBM Corp., Armonk, NY, USA). Due to the small number of recorded WISE sessions, we could not utilize inferential statistics to compare fidelity scores between settings. Rather, we used descriptive statistics consisting of means (*M*) and standard deviations (*SD*s) of the WISE fidelity sum score for each setting.

### 2.3. Research Question 2

What are stakeholders’ perceptions of: (a) acceptability, (b) appropriateness, (c) feasibility, and (d) perceived sustainability of the WISE curriculum in a single generation and intergenerational setting? We utilized quantitative and qualitative methods to answer this question.

#### 2.3.1. Quantitative Method

The research team explored an indirect assessment of stakeholder perceptions of WISE acceptability, appropriateness, and feasibility within single generation and intergenerational settings using the implementation outcomes measures developed by Weiner and colleagues [36]. Stakeholders (i.e., preschool teachers and site staff) who observed at least one WISE session were asked to complete the measures.

#### 2.3.2. Measures

The authors used 4-item measures of intervention acceptability, appropriateness, and feasibility [36]. Stakeholder perceptions were captured with Likert scale items rated from one (Completely Disagree) to five (Completely Agree). We calculated an item average for each scale to evaluate the three implementation dimensions separately, with higher scores indicating greater levels of acceptance, appropriateness, or feasibility. Cronbach’s alpha ranged from 0.94 for the Appropriateness of Intervention Measure to 1.00 for the Acceptability of Intervention Measure.

#### 2.3.3. Analysis

Data were analyzed using SPSS version 25 (IBM Corp. Released 2017. IBM SPSS Statistics for Windows, Version 25.0. Armonk, NY: IBM Corp.). Descriptive statistics consisting of individual means (*M*) and standard deviations (*SD*s) summarized stakeholders’ perceptions of acceptability, appropriateness, and feasibility of the WISE curriculum.

##### Directed Qualitative Content Analysis Method

We utilized a deductive approach of directed qualitative content analysis [37] to understand stakeholder perceptions of WISE based on four implementation outcomes. Additionally, we followed the 16 suggested directed qualitative content analysis steps provided by Assarroudi and colleagues [38] to code interview/focus group transcripts (*n* = 4) and detailed field notes (*n* = 2) from annual interviews/focus groups conducted with consented stakeholders (i.e., FFLL team members and site staff) by the FFLL research team in the summer of 2020 and 2021. We will highlight the most salient steps used in the current study. Interviews/focus groups addressed implementation with questions such as: “What was your experience delivering or observing the WISE curriculum?” and “What have you observed in your participants since integrating [implementing] the WISE curriculum [within both a single generation and intergenerational setting]?” Transcripts and field notes were chosen as the unit of analysis if the stakeholder(s) commented on the implementation outcomes of interest regarding WISE within their interview/focus group (step 6) [38]. One author highlighted segments of interview transcripts and field notes where implementation outcomes were discussed to guide coding. The four assessed implementation outcomes (i.e., acceptability, appropriateness, feasibility, and perceived sustainability) served as preliminary category codes to develop a formative categorization matrix (step 8) [38] that guided pre-testing and then main data analysis in the directed qualitative content analysis. Then, in accordance with step 9, we identified theoretical definitions of these categories from Proctor and colleagues’ evaluation framework [27] (see Table 2).

To establish interrater agreement, two coders first individually read and coded highlighted sections of two interview transcripts in Atlas.ti (Version 9, Scientific Software Development GmbH, Berlin, Germany) using the four category codes and making notes in memos and using quotations to conduct inductive abstraction to obtain the essence of each code (step 14) [38]. Next, the coders discussed their category coding and inductive abstraction that grouped preliminary codes into ‘generic categories’ until coming to 100% consensus.

Upon reaching adequate interrater agreement, the remaining four transcripts and focus group notes were coded. The coders met a final time to review codes, generic and main categories, and inductive abstraction until 100% consensus was achieved. Last, the coders established links between the categories to complete the final phase of reporting (step 15) [38].

## 3. Results

### 3.1. Research Question 1

#### Quantitative Findings

In total, six single generation and three intergenerational video-recorded WISE sessions were analyzed. These represented lessons on target foods (i.e., berries, carrots, green peppers, and tomatoes) delivered among four different preschool classrooms at two preschools (two participating in only single generation WISE and two participating in single generation and intergenerational WISE programming) and one adult day services center. One video (intergenerational setting) was excluded from analysis because the trained WISE facilitator did not deliver the programming. The recorded sessions were on average 31 min and 45 s long. Results indicated that fidelity scores were comparable between single generation (*M* = 3.45, *SD* = 0.18) and intergenerational (*M* = 3.30, *SD* = 0.12) settings.

### 3.2. Research Question 2

#### 3.2.1. Quantitative Findings

Nine stakeholders completed the acceptability, appropriateness, and feasibility measures. However, the final sample consisted of six stakeholders due to missing data (*n* = 1), consent refusal (*n* = 1), and an outlier (*n* = 1). Five of the six stakeholders represented one preschool. Stakeholders included five teachers and one administrator. Overall, findings revealed that stakeholders’ perceptions of the appropriateness (*M* = 4.79, *SD* = 0.400), feasibility (*M* = 4.46, *SD* = 0.84), and acceptability (*M* = 4.83, *SD* = 0.41) of WISE were high.

#### 3.2.2. Directed Qualitative Content Analysis Findings

Interviews and focus groups included 12 stakeholders representing preschool teachers (*n* = 6), preschool administrators (*n* = 2), adult day center staff (*n* = 2) and FFLL team members (*n* = 2).

##### Acceptability

Overall, stakeholders expressed that they liked WISE programming and perceived certain components to be acceptable for both preschool and older adult participants. They expressed support for the program’s implementation. For example, an FFLL team member shared, “I really enjoyed the WISE curriculum, I think [WISE] was a perfect fit for us, and what we were looking for … I really enjoyed learning [WISE] and implementing it.” Similar feedback was received from a preschool administrator who said, “The feedback I have gotten is that [the preschool teachers] really liked WISE. I think that was probably their favorite curriculum.” Similarly, adult day center staff expressed disappointment that intergenerational WISE programming discontinued due to the pandemic, saying, “I hate that we couldn’t explore it any further than we did ‘cause [FFLL team member] was really doin’ a good job with it, and I think it would have continued to grow.” A FFLL team member recalled adult day center stakeholders asking, “‘When are y’all coming back; when can we get our [intergenerational programming] back, and when can the preschoolers come back?’” As demonstrated, the intergenerational WISE programming strengthened interest from adult day center staff to incorporate more intergenerational opportunities.

Child and older adult participants were drawn to different elements of WISE. Children were excited to see the WISE mascot, Windy the Owl, whereas older adults expressed favorable sentiments toward the intergenerational nature of programming and trying new foods and recipes. Adult day center staff stated, “I think part of it was—the biggest part—was the children being here, but the other part was—[older adult participants] did enjoy trying the new snacks and dips and things, recipes and stuff that we gave to them that [the FFLL team member] brought to the program.” Interestingly, older adults demonstrated increased interest in intergenerational programming over time with more older adults participating as they became familiar with the program, as noted by a FFLL team member, “When you’ve got people [older adults] that are fighting to get in that room the minute they see [the FFLL team member], I think that speaks for itself.”

##### Appropriateness

Importantly, stakeholders, including preschool stakeholders, agreed that WISE was appropriate for the preschoolers. One administrator commented, “…the target foods [curriculum], I think, [WISE] was a little more specific—it seemed very intentional as opposed to some of the other curriculums {sic} [where] you’re doing this book, this book, this book [other curricula]. [WISE] seemed more intentional to nutrition.” An FFLL team member commented on how WISE in the intergenerational setting helped fill a gap in children’s development, explaining, “[WISE] gave an experience [with intergenerational connections] in addition to the nutrition education … it gave those kids a life experience to have that [intergenerational] relationship that they may not ever have, and I think that was a huge success.” While older adult participants responded favorably to intergenerational sessions that included recipes and food tastings, adult day center staff noted that the older adults may not have grasped the nutrition information. For example, one adult day center staff member reported, “… I’m not for sure that my participants really picked up so much on the healthy food initiative … they just enjoyed the interaction with the children, which was still good, it was still positive.”

The adult day center staff also mentioned the appropriateness of program novelty in setting the intergenerational nutrition program schedule; as one staff member stated, “if you tried to do [WISE intergenerational programming] more [than once a month], it would have become redundant, and you would have lost their attention.”

While stakeholders noted many aspects of WISE that were appropriate, they also shared aspects that were less appropriate. Though stakeholders agreed that including Windy the Owl was appropriate and enjoyed by the children, they felt it was not appropriate for older adults. For instance, an adult day center staff said, “I don’t think [the older adults] felt like [Windy the Owl] was childish, but I don’t think they understood … what Windy was trying to do.” The FFLL team member who led programming shared a similar perspective, reporting “I did feel as though the puppet with the older adults wasn’t as appropriate [as it was for children],” and she described adapting Windy’s role in the intergenerational setting by saying “I brought [Windy] here today and she gets to see her grandfriends” like the children do. Additionally, preschool stakeholders and FFLL team members noted that certain WISE components proved challenging in the roving model for younger preschool participants’ attention span, such as the amount of time it takes for children to listen to the letter from a farmer about the target food.

##### Feasibility

Stakeholders discussed ways that intergenerational WISE programming can accommodate noted barriers. Specifically, stakeholders emphasized challenges of sharing physical space, commenting that a large room is desirable to accommodate high interest among participants. Additionally, a preschool administrator shared difficulties with transportation to sites, which may be ameliorated with additional resources. A FFLL team member commented that activity leaders should receive WISE and intergenerational program training to ensure feasibility. Both preschool and adult day center stakeholders discussed the importance of forming partnerships for future intergenerational collaboration. One adult day center stakeholder communicated the need for continued partnerships with preschools once COVID-19 restrictions eased:

Well, we would just need to reconnect, and then- I would have to make a conscious effort to put [intergenerational programming] on my calendar to just reach out from time to time and say, ‘hey, I’m still here,’ ‘are y’all doing anything that I might be a part of?’

Barriers regarding population characteristics were also discussed. For some younger children, the roving model’s two monthly sessions may not have provided adequate time to comprehend curricular elements, such as needed reminders about the target food before programming activities began. Participants also discussed the feasibility of the WISE format. The FFLL team member who delivered WISE noted challenges in the roving model. They specified:

Pre-COVID, [delivering the curriculum] was tricky, I think the recipes were tricky to incorporate … ‘cause we were a roving model … so that was a little bit harder incorporate more depth into the lessons because … we were the ones moving to children and adults, and during COVID [when] we pivoted to [a new preschool], the children and teachers came to me, so I was able to set up and do more because I wasn’t having to pack up and move.

Several stakeholders, especially teachers, noted that WISE could be feasibly implemented within a virtual setting to continue nutrition education during the COVID-19 pandemic, which some teachers delivered to their students over Zoom.

##### Perceived Sustainability

Barriers noted in the feasibility section above have a direct impact on program sustainability. For instance, preschool and adult day center stakeholders communicated the potential for WISE programming to continue despite COVID-19 challenges. A FFLL team member emphasized that, “… we [FFLL] went ahead and offered the WISE training for [the preschool teachers] during COVID-19 so the teachers and families … could continue to connect virtually” in a single generation setting without the FFLL team member. In fact, one preschool administrator mentioned that certain preschool teachers did continue virtual single generation programming with their students after receiving training. Additionally, stakeholders expressed interest in the continuation of single generation and intergenerational WISE programming beyond FFLL funding.

Regarding intergenerational WISE programming, the adult day center stakeholder mentioned similar barriers of program sustainability to program feasibility. Among older adult stakeholders, respondents shared that consistent participation by older individuals may be difficult given life events the population frequently experiences. Such observations were also made by some preschool teachers regarding their students’ ability to complete the WISE curriculum. To exemplify, one stakeholder said, “’cause our population is very fluid; they may be here for three months, and then they disenroll for some reason, we don’t see them again, and then we have new people come in …”.

Stakeholders also mentioned needing to make connections with other preschool sites post-COVID-19 for intergenerational programming opportunities to continue. Despite these challenges, an adult day center staff member noted that, “I really felt like [WISE] was beginning to take off when we had to shut it down … we had momentum… as far as … getting participants going to it and stuff.” Enthusiastic optimism for resuming intergenerational WISE programming provided support for its potential sustainability within the community setting.

## 4. Discussion

Extant research has documented the positive impacts intergenerational programming can have on participants [24,39]. However, intergenerational research rarely applies implementation science to investigate performance gaps in programming and how to scale-up community intergenerational programs [40]. The evidence-based WISE curriculum has been associated with increasing vegetable and fruit intake among preschoolers [15,16], yet research had not evaluated its implementation within the intergenerational setting. As people of varied ages need care, research focusing on implementation programs such as WISE in intergenerational settings can benefit all those participating [41]. Our study utilizes multiple data sources to address two research questions aimed at assessing and comparing implementation of the WISE curriculum within single generation and intergenerational settings.

Findings from the quantitative direct and indirect assessments indicate that fidelity of WISE programming is comparable within single generation and intergenerational settings. Additionally, acceptability, appropriateness, and feasibility of the WISE curriculum are rated high overall among key stakeholders. The qualitative findings from the directed qualitative content analysis reinforce the quantitative ratings while highlighting common barriers in intergenerational program research, including logistical considerations and the need for strong community partnerships [39,42]. Overall, results from this study provide support for the use of WISE within an intergenerational setting when considerations are taken to ensure both age groups can experience benefits the program offers.

Stakeholders representing older adult and preschool care settings found WISE to be acceptable. Feasibility and sustainability scores overlap; if WISE is not feasible, it may not be sustainable. Certain aspects of the curriculum may require adaptation to fit the needs of intergenerational participants, which is common and important for tailoring interventions for different contexts [43]. For example, modifications to introduce and incorporate Windy the Owl to engage preschoolers can address concerns about age-appropriateness for older adults [44]. In addition, ensuring older adults gain WISE nutrition education in additional formats could enhance acceptability of the curriculum within an intergenerational setting. Finally, determining a practical program model for intergenerational settings requires exploration of the roving and stationary models. A stationary model may be achieved if the children’s and older adults’ programs are co-located; WISE may be partially stationary if, for example, a preschool teacher is trained to deliver WISE, and the older adult participants travel to the preschool for shared programming.

### 4.1. Fidelity of WISE Using an Intergenerational Approach

Although WISE program fidelity has been assessed in a classroom setting [28,29], this is the first study to examine its fidelity in an intergenerational setting. As results indicate, scores are comparable across settings suggesting that trained facilitators could expand WISE into intergenerational settings to achieve results comparable to those in a single generation classroom setting. As WISE programming specifies modeling behaviors from teachers, older adults could also model behaviors [4], such as tasting and making positive comments to the children about the target food.

### 4.2. Stakeholder Acceptance and Appropriateness of an Intergenerational Approach to WISE

Ratings of WISE acceptability and appropriateness are high, with respondents providing a favorable evaluation of every item comprising the three scales. Lower ratings would have indicated a need to refine the implementation protocol for the focal audience, including the intergenerational component [14]. Through qualitative analysis, preschool and older adult stakeholders enjoyed and accepted WISE with participants showing interest in programming, prioritizing WISE in programming, and having administrative support to pursue an intergenerational WISE program. The educational component was more appropriate for preschoolers than older adults; the intergenerational aspect of WISE was, however, deemed appropriate for both older adults and preschoolers. Evaluation of the curriculum as acceptable and appropriate is critical if sustainability beyond the grant funding period is desired [27].

### 4.3. Feasibility and Program Sustainability

Barriers to program feasibility emerged during qualitative interviews and focus groups, indicating that not all aspects of WISE can be incorporated within an intergenerational setting. As such, findings point to a potential decreased likelihood of intergenerational program sustainability if feasibility concerns are not addressed, despite wishes from stakeholders for programming to continue. Adaptations can be continuously made to meet the needs of the participants and environmental situations (e.g., COVID-19 restrictions). Importantly, findings indicate a willingness to persevere during challenging circumstances, with some teachers continuing to deliver WISE virtually during COVID-19 and other preschool teachers committing to the program by completing WISE training. However, stakeholders recognize the importance of community support for intergenerational sessions to continue past the grant funding period.

### 4.4. Strengths and Implications

Although the implementation of intergenerational programming is a novel approach to nutrition education, intergenerational programming has existed in other domains and has its own set of intergenerational program best practices [45,46]. Several WISE fidelity indicators parallel intergenerational best practices, further illustrating the strengths of intergenerational nutrition programming. For example, the fidelity indicator of actively involving children in WISE programming aligns with the intergenerational best practice of providing older adults with appropriate and meaningful roles, which has been associated with achieving program goals [47]. Where the WISE fidelity measure emphasizes trying new foods, intergenerational best practices emphasize promoting intergenerational interaction. Both indicators rely on trained facilitators to support programming goals. Furthermore, this study illustrates that preschool nutrition curricula can be implemented with another age group potentially achieving benefits beyond those of single generation programming. Still, one should proceed with caution to avoid infantilizing older adults [44,48]. Where WISE values using Windy the Owl to interact with the children, intergenerational best practices would suggest age-appropriate roles, such as inviting older adults to help facilitate the imaginative inclusion of the puppet. WISE provides other opportunities for various levels of age-appropriate participation, including observing or encouraging the children during the learning process.

Implications of our findings can translate into action. As evidenced by acceptability measure results, staff at FFLL sites welcome the inclusion of intergenerational practices in their curriculum. Other preschool educators could consider providing an intergenerational component of WISE programming to provide additional models of positive behaviors. With an intergenerational community partner, preschools could expand their nutrition programming to include older adults, potentially benefiting both age groups. Benefits of shared interaction among preschool students and older adults during nutrition programming could include transfer of knowledge and shared pleasant experiences [21].

### 4.5. Limitations and Future Research

First, WISE programming was delivered using a roving model. This model led to lessons being delivered less frequently to entire classrooms and in an intergenerational setting, in contrast to the recommended delivery of weekly lessons to small groups within a class delivered by the students’ teacher [14]. Some of the necessary changes to programming resulted in lower fidelity ratings on the item “completes in prescribed group size.” The use of video recording was another limitation. At times, participants moved out of the camera frame, making coding difficult; authors complemented visual with auditory cues. Despite being a pilot study, our sample size was notably small for both the number of videos and stakeholders. Our video sample size was particularly small due to program discontinuation required because of COVID-19 social distancing restrictions. Finally, we cannot conclude that stakeholders’ ratings of WISE acceptability, appropriateness, and feasibility were explicitly derived from observing programming in an intergenerational setting. Regarding our qualitative data, the categories used to guide the directed content analysis were not mutually exclusive [27], resulting in quotes earning more than one code from our categorization matrix. Although interviews were transcribed, focus group field notes did not include direct quotes, potentially limiting interpretation.

Based on our findings, future research should replicate the present study with a larger sample to determine if fidelity scores differ significantly between the two settings. Similarly, a larger sample would allow researchers to explore differences of WISE acceptability, appropriateness, and feasibility between stakeholder groups. Last, researchers should test whether WISE program outcomes are equally effective in an intergenerational setting to investigate how older adults influence nutritional outcomes.

## Figures and Tables

**Table 1:** WISE 18-item Fidelity Measure ^1^.

Item	Not at All	Somewhat	Quite a Bit	Very Much
Completes in prescribed group size	Whole class	Groups of 8+	Groups 6–8	Groups 4–6
Emphasizes trying	0 times	1 time	2–3 times	4+ times
Involves children as prescribed (See manual for specifics)	No children had roles	Less than half of the children had a role	More than half of the children had a role	Every child had a role at some point
Eats target food	Did not try food at all	Tried food with 1 group	Tried food with most groups	Tried food with all groups
Positive comments	0	1	2–3 times	4+ times
Uses Windy during activity	Not at all	Mentions Windy but does not use the puppet	1–2 times during activity	Windy is an integral part of activity
Leads class in Windy’s ‘Whooo tried it?’ chant	Does not complete chant	Completes chant without Windy	Holds Windy during class chant	Uses Windy and completes chant with enthusiasm
Seems prepared	Has no supplies on hand	Has some supplies on hand	Has most supplies on hand	Has all supplies and materials on hand
Paces lesson appropriately ^2^	Most children experience long waits or feel rushed	Some children are rushed or experience long waits	Few children are rushed or experience long waits	Time managed well—no long waits or rushing
Responds to questions/comments	Not at all or in a clipped or inattentive way	Is attentive to and responds well to only select children	Is attentive to and responds well to most children	Is attentive to and responds well to all children
Give/maintain engagement	No control of classroom behavior for >50% of the lesson	No control of classroom behavior 25–50% of the lesson	Maintains control of classroom behavior 50–75% of the lesson	Uses counting, songs, or transition activities to engage children when needed
Negative comments about target food ^3^	0	1	2–3 times	4+ times
Uses Windy inappropriately ^3^	0	1	2–3 times	4+ times
Threatens or forces target food ^3^	0	1	2–3 times	4+ times
Encourages talk with parents	Does not mention the home, family, or parents	Mentions family without connecting the food and home	Suggests that they try the recipe at home	Directly asks or encourages children to talk about target food with family
Seems comfortable using Windy	Does not use Windy	Windy is put on the teacher’s hand but not used as a character	Windy is involved but only whispers to teacher	Windy has a voice and participates
Children seem engaged ^2^	Most children are distracted and/or engaging in other behaviors	Engaged 25% of the time	Engaged >25% of the time	All children are focused on the lesson

^1^ Adapted from [28]; ^2^ When coding this response, the activity was thought of as a whole by coders. ^3^ This item was reverse coded, so that a lower score indicated greater fidelity.

**Table 2:** Category definitions and anchor examples for the directed qualitative content analysis.

Category Codes	Definition	Anchor Examples
Acceptability	The perception among implementation stakeholders that a given treatment, service, practice, or innovation is agreeable, palatable, or satisfactory.	“The feedback I have gotten is that [the preschool teachers] *really* liked WISE. I think that was probably their favorite curriculum.”
Appropriateness	The perceived fit, relevance, or compatibility of the innovation or evidence-based practice for a given practice setting, provider, or consumer; and/or perceived fit of the innovation to address a particular issue or problem.	“[WISE] gave an experience [with intergenerational connections] in addition to the nutrition education. It gave those kids a life experience to have that [intergenerational] relationship that they may not ever have, and I think that was a huge success.”
Feasibility	The extent to which a new treatment, or an innovation, can be successfully used or carried out within a given agency or setting.	“Well, we would just need to reconnect, and then—I would have to make a conscious effort to put [intergenerational programming] on my calendar to just reach out from time to time and say, ‘hey, I’m still here’, ‘are y’all doing anything that I might be a part of?’”
Perceived Sustainability	The perception that the newly implemented treatment can be maintained or institutionalized within a service setting’s ongoing, stable operations.	“…cause our population is very fluid; they may be here for three months, and then they disenroll for some reason, we don’t see them again, and then we have new people come in…”

Note: Definitions of acceptability, appropriateness, and feasibility were directly derived from Proctor et al.’s [27] evaluation framework (pp. 67–70). The definition of the category “perceived sustainability” was adapted from Proctor et al. [27] to fit the context of the present study. Exemplar quotes are provided for each category code.

## Data Availability

Some or all data and models that support the findings of this study are available from the corresponding author upon reasonable request.
